# Using Electronic Medical Record Data for Research in a Healthcare Information and Management Systems Society (HIMSS) Analytics Electronic Medical Record Adoption Model (EMRAM) Stage 7 Hospital in Beijing: Cross-sectional Study

**DOI:** 10.2196/24405

**Published:** 2021-08-03

**Authors:** Rui Li, Yue Niu, Sarah Robbins Scott, Chu Zhou, Lan Lan, Zhigang Liang, Jia Li

**Affiliations:** 1 Information Center Xuanwu Hospital Capital Medical University Beijing China; 2 Statistical Procedure Department Blueballon (Beijing) Medical Research Co, Ltd Beijing China; 3 National Center for AIDS/STD Control and Prevention Chinese Center for Disease Control and Prevention Beijing China; 4 West China Biomedical Big Data Center West China Hospital Sichuan University Beijing China

**Keywords:** electronic medical records, data utilization, medical research, China

## Abstract

**Background:**

With the proliferation of electronic medical record (EMR) systems, there is an increasing interest in utilizing EMR data for medical research; yet, there is no quantitative research on EMR data utilization for medical research purposes in China.

**Objective:**

This study aimed to understand how and to what extent EMR data are utilized for medical research purposes in a Healthcare Information and Management Systems Society (HIMSS) Analytics Electronic Medical Record Adoption Model (EMRAM) Stage 7 hospital in Beijing, China. Obstacles and issues in the utilization of EMR data were also explored to provide a foundation for the improved utilization of such data.

**Methods:**

For this descriptive cross-sectional study, cluster sampling from Xuanwu Hospital, one of two Stage 7 hospitals in Beijing, was conducted from 2016 to 2019. The utilization of EMR data was described as the number of requests, the proportion of requesters, and the frequency of requests per capita. Comparisons by year, professional title, and age were conducted by double-sided chi-square tests.

**Results:**

From 2016 to 2019, EMR data utilization was poor, as the proportion of requesters was 5.8% and the frequency was 0.1 times per person per year. The frequency per capita gradually slowed and older senior-level staff more frequently used EMR data compared with younger staff.

**Conclusions:**

The value of using EMR data for research purposes is not well studied in China. More research is needed to quantify to what extent EMR data are utilized across all hospitals in Beijing and how these systems can enhance future studies. The results of this study also suggest that young doctors may be less exposed or have less reason to access such research methods.

## Introduction

Electronic medical records (EMRs), or digitized versions of patient medical charts, are often considered a key component of a hospital or health care system’s health information system [1]. EMR systems have transformed data and record keeping in the medical field, and they enable providers to more systematically track patient information over time, promote a more holistic approach to patient care, support the streamlining of preventative screening, support the monitoring of patients, and improve overall quality [2,3]. For these reasons, there has been rapid growth in the implementation of EMR systems in health care settings throughout the world in recent decades [4-9]. Subsequently, the amount and availability of clinical data automatically collected by EMRs are increasing at an exponential rate [10,11], and EMRs have been recognized as a valuable resource for observational data and for large-scale analyses [12,13]. As such, EMR data are often used for research purposes in many universities and organizations around the world [14,15]. Using EMR data for medical research [16,17] has several benefits, such as being low cost, having a large volume of data, and saving time because there is no need to recruit and retain participants [18-21]. Thus, it is believed that using EMRs to obtain clinical information has the potential to revolutionize medical research in the coming years [22,23].

In China, the EMR system has become the core system for the collection and management of hospital information, as the National Electronic Medical Record System has been promoted across the country since 2011 [24-26]. Furthermore, with many hospitals implementing the Healthcare Information and Management Systems Society (HIMSS) Analytics Electronic Medical Record Adoption Model (EMRAM) standards, numerous Chinese hospitals have become international standard and accredited hospitals [27]. One result of this shift has been that increasing numbers of western institutions are collaborating with China on medical research using EMR data [28].

As research using EMR data has become increasingly prevalent, researchers have been pondering how to better explore the technical value of EMR data. In addition, there exists a growing body of literature on the feasibility and efficacy of using electronic health records for research purposes. Electronic health records (EHRs) are inclusive of a broader view of patient care, including diagnoses, medications, immunizations, family medical history, and provider contact information. EMR data, however, are digital versions of patient charts. They contain notes and information collected by and for clinicians in that particular care setting and are mostly used by providers for diagnosis and treatment [3]. In China and abroad, studies on the topic of using EMR or EHR data for research have primarily focused on the challenges of using such systems. Researchers over a decade ago raised concerns regarding the quality and comprehensiveness of clinical data being collected in EMR systems and mentioned that there were systematic biases inherent to data collected primarily for clinical care [29]. Other studies have identified other barriers, including legal, technical, ethical, social, and resource-related issues, such as privacy protection, data security, data custodians, and the motives for collecting data, as well as a lack of incentives to share data [15,30]. An additional systematic review identified four domains of potential limitations, including data quality issues (91.7%), data preprocessing challenges (53.3%), privacy concerns (18.3%), and potential for limited generalizability (21.7%) [31]. Some studies have consequently developed a list of caveats and recommendations for overcoming such limitations [30,32-35].

Additionally, the majority of existing research focuses on the quality of EMR/EHR data and its related challenges [36-39]. These challenges can be divided into five primary areas as follows: completeness, consistency, validity, reliability, and accuracy [40-42]. Some analyses have aimed to develop assessment frameworks to ensure data quality across studies [43], but there are few studies that quantitatively explore how and to what extent EMR or EHR data are being collected and used in China. Thus, it is necessary to build EMR data quality metrics and standardize routine documentation to enable its secondary use for medical research [44-46].

The paralleled use of EMR data for medical research has been noted. In one such study, the characteristics of EMR data in China were compared against data collected in hospitals in the United States in order to understand system and cultural differences that may exist between Chinese and English clinical documents [47]. A study by van Velthoven et al [48], for example, shed light on the feasibility of extracting EMR data across a number of countries. These studies are useful for understanding how data collection systems in China and the use of EMR data for medical research may adapt to more international standards, further supporting collaboration between Chinese and foreign research institutions.

Currently, in Chinese hospitals, the data available to researchers are limited in scope to just EMRs, rather than full EHRs. In order to further promote utilizing EMR data for research, a quantitative investigation of the current status of data utilization is warranted, since understanding the status quo is a prerequisite for determining barriers and improving the existing system. It is necessary to explore the obstacles that hinder EMR data utilization for medical research from the perspective of data consumers, but there is currently no quantitative research or surveys published on the recent status of EMR data utilization for medical research in any institution or region in China. Thus, this study aimed to understand the landscape, including barriers and obstacles, of utilizing EMR data for medical research in Chinese medical institutions. This study will provide data managers and medical research managers with a broader understanding of what types of data are being used; what extent they are being utilized; and who is accessing such data, laying the groundwork for further promotion of this research method.

## Methods

### Study Design

A serial, cross-sectional, descriptive study was carried out at Xuanwu Hospital, Capital Medical University (XWHCMU) in Beijing, China. XWHCMU is a large 1600-bed tertiary general hospital with a complete EMR data repository and is one of the two HIMSS Analytics EMRAM Stage 7 hospitals in Beijing. The HIMSS Analytics EMRAM incorporates methodology and algorithms to automatically score hospitals around the world relative to their EMR capabilities. A Stage 7 rating signifies the highest level of EMR function and application, achieving a near paperless environment that harnesses technology to support optimized patient care. At Xuanwu Hospital, the EMRAM data system was implemented in 2014. All employees receive training on the content and scope of the EMR data available, the permissions for EMR data utilization, and the process of requesting and obtaining EMR data.

### Data Sources and Extraction

All data from the Office Information System (Office Automation) was extracted, because each EMR data extraction request in the hospital must be approved through the EMR data management module in the Office Automation. Variables of interest included data request purpose, requester ID, requester department, and data request time. If the purpose of the data request was for scientific research, it was included in the study. The requester ID was used to retrieve the age and professional title of all requesters in the hospital human resources dictionary. The requester ID was also used as the main index for data matching and integration, forming a total of 933 EMR data request records for scientific research purposes between 2016 and 2019.

The use of EMR data for research purposes by key departments in the hospital was also assessed. XWHCMU evaluates the scientific research performance of each department every year based on a set of 18 evaluation criteria, including published papers/books, transformation of scientific research results, academic events, and approved scientific research projects. The top 10 clinical departments with the highest cumulative research work performance score over the last 4 years were selected as “key departments” for this study. The performance score of each department, evaluation indicators, and standards of scientific research work can be found in Multimedia Appendix 1.

### Statistical Analysis

The data were analyzed using IBM SPSS Statistics for Windows version 23.0 (IBM Corp). The data were expressed using times, frequencies, and percentages. The chi-square test was used for categorical variables, with *P*<.05 considered statistically significant. A summary of the statistical indicators, their definitions, and how they were calculated can be found in Table 1.

**Table 1:** Summary of the statistical indicators of the study, their corresponding definitions, and how they were calculated.

Statistical indicators	Definition	Calculation
Times	An absolute value index	The cumulative value of the number of requests for electronic medical record (EMR) data for research by professional and technical personnel in the observation unit (institution or department) during the observation period.
Frequency	An intensity index	The number of requests/∑the number of professional and technical personnel in this observation unit × time.
Proportion of requesters	A ratio indicator	∑the number of professional and technical personnel who have requested EMR data for research/∑the number of professional and technical personnel in this observation unit.
Number of departments that did not request data	A counting indicator	The number of departments that never requested EMR data for scientific research during the observation period.
Absolute increment of frequency	The absolute value of growth	Can be further divided into cumulative growth and annual growth.
Cumulative growth	The absolute value of growth	The difference between the frequency of a certain year and that at baseline (2016).
Annual growth	The absolute value of growth	The difference between the frequency of a year and that of the previous year.
Frequency growth rate	The growth rate of frequency	Divided into fixed base ratio growth rate and link ratio growth rate.
Relative ratio with fixed base	The growth rate of frequency	The net increase rate of frequency in a certain year compared with the baseline (2016), that is, the ratio of a certain year’s frequency to the baseline frequency minus 100%.
Link relative	The growth rate of frequency	The net increase rate of frequency in a year compared with the frequency of the previous year, that is, the ratio of frequency of a year to that of the previous year minus 100%.

## Results

### EMR Data Utilization From 2016 to 2019 at XWHCMU

The frequency of EMR data utilization increased from 0.06 times per person per year (2016) to 0.1 times per person per year (2019), and the proportion of requesters increased from 3.3% (2016) to 5.8% (2019), as seen in Table 2. The majority of medical departments at the hospital are using the EMR system, with the number not using the system decreasing from 21 (2016) to 5 (2019). The fixed base ratio growth rate of the frequency of EMR data utilization was 66.67%, and the year-to-year growth rate in 2019 was zero.

The frequency at which EMR data was used for medical research increased significantly between 2016 and 2018 (Table 2). The growth rate frequency has gradually slowed down over the past 4 years, with a bottleneck occurring in 2019, during which the growth rate was 0%.

**Table 2:** General trends in the utilization of electronic medical records in Xuanwu Hospital, Capital Medical University, Beijing, China between 2016 and 2019.

Year	Times	Frequency	Proportion of requesters, n/N (%)	Number of departments that did not request data, n/N (%)	Absolute increment of request frequency			Request frequency growth rate, %	
					Cumulative growth	Annual growth	Relative ratio with fixed base		Link relative
2016	171	0.06	98/3060 (3.2%)	21/47 (44.7%)	N/A^a^	N/A	N/A		N/A
2017	201	0.07	119/2935 (4.1%)	19 /47 (40.4%)	0.01	0.01	16.67		16.67
2018	288	0.10	153/2883 (5.3%)	14/47 (29.8%)	0.04	0.03	66.67		42.86
2019	273	0.10	163/2667 (6.1%)	5/47 (10.6%)	0.04	0.00	66.67		0.00

^a^N/A: not applicable.

### Utilization of EMR Data by Key Departments at XWHCMU From 2016 to 2019

The key departments had a per capita request frequency lower than the average per capita request frequency for the overall hospital (Table 3). The proportion of data utilization by key departments decreased from 70.0% in 2016 to 49.4% in 2019.

**Table 3:** Utilization of electronic medical record data in the key scientific research departments of Xuanwu Hospital, Capital Medical University, Beijing, China between 2016 and 2019.

Research score ranking	Department	2016				2017				2018				2019		
		Times	Proportion of the whole hospital request times, %	Frequency	Times		Proportion of the whole hospital request times, %	Frequency	Times		Proportion of the whole hospital request times, %	Frequency	Times		Proportion of the whole hospital request times, %	Frequency
1	Neurology	49	28.8%	0.16	57		28.4%	0.19	70		24.3%	0.23	65		23.8%	0.16
2	Neurosurgery	18	10.6%	0.08	17		8.5%	0.08	16		5.6%	0.07^a^	8		2.9%	0.03^a^
3	Radiology	7	4.1%	0.06	6		3.0%	0.05^a^	8		2.8%	0.07^a^	7		2.6%	0.06^a^
4	General Surgery	5	2.9%	0.05^a^	21		10.4%	0.20	16		5.6%	0.15	10		3.7%	0.07^a^
5	Functional Neurosurgery	1	0.6%	0.01^a^	1		0.5%	0.01^a^	4		1.4%	0.05^a^	4		1.5%	0.05^a^
6	Interventional Radiography	0	0%	0.00^a^	1		0.5%	0.03^a^	9		3.1%	0.26	5		1.9%	0.14
7	Vascular Surgery	13	7.7%	0.19	15		7.5%	0.22	13		4.5%	0.19	5		1.9%	0.07^a^
8	Anesthesiology	0	0%	0.00^a^	2		1.0%	0.01^a^	2		0.7%	0.01^a^	6		2.0%	0.03^a^
9	Pharmacy	25	14.7%	0.20	19		9.5%	0.15	33		11.5%	0.26	22		8.1%	0.17
10	Orthopedics	1	0.6%	0.02^a^	3		1.5%	0.05^a^	3		1.0%	0.05^a^	3		1.0%	0.05^a^
Total	N/A^b^	119	70.0%	N/A	142		70.8%	N/A	174		60.5%	N/A	135		49.4%	N/A
Frequency of the overall hospital	N/A	N/A	N/A	0.06	N/A		N/A	0.07	N/A		N/A	0.10	N/A		N/A	0.10

^a^The annual per capita electronic medical record data utilization frequency of this department was lower than the annual average of the whole hospital. The annual average is based on all departments.

^b^N/A: not applicable.

### Utilization of EMR Data by Age

**Figure 1:**
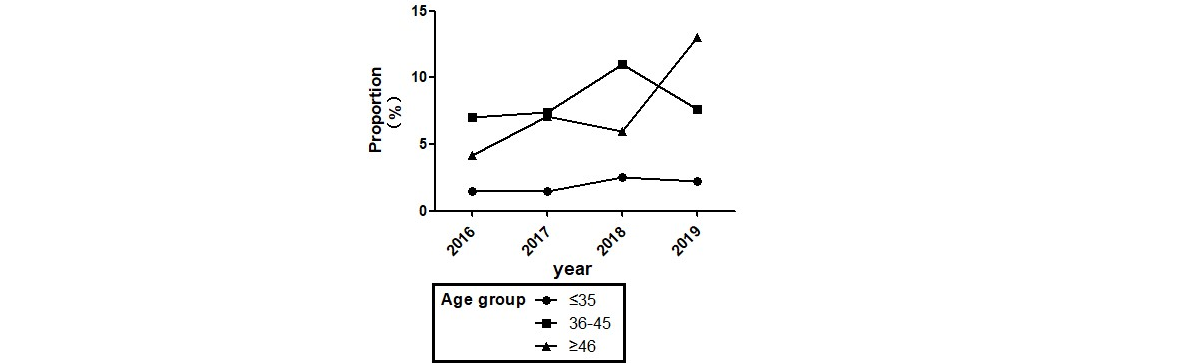
Trend in the proportion of electronic medical record data users by age group at Xuanwu Hospital, Capital Medical University, Beijing, China between 2016 and 2019.

As seen in Figure 1, the trend in the proportion of individuals using EMR data varied between 2016 and 2019. Those aged 36 to 45 years made up the largest proportion of researchers using EMR data from 2016 to 2018, though this trend declined in 2019, when those aged 46 years of age or older made up the larger proportion of requests. Generally speaking, those under the age of 35 years represented the smallest proportion of EMR data users at the hospital.

### Utilization of EMR Data by Staff Level

In 2016, the proportion of junior-level professionals using EMR data for medical research was the lowest (1.2%), while those with senior-level titles made up the largest proportion of EMR data users (8.8%). This trend continued through 2019, as seen in Table 4. Between 2016 and 2019, senior-level professionals made up the largest proportion of those requesting EMR data (255/533, 47.8%), followed by intermediate-level staff (161/533, 30.2%) and then junior-level staff (117/533, 21.9%). Over the 4-year period, the proportion of senior- and intermediate-level staff requesting EMR data increased, while there was no significant change in the junior-level staff group.

**Table 4:** Electronic medical record data utilization by junior-, intermediate-, and senior-level staff at Xuanwu Hospital, Capital Medical University, Beijing, China between 2016 and 2019.

Year	Professional title				Total, n/N (%)		Chi-square (*df*)		*P* value
	Junior-level requester, n/N (%)	Intermediate-level requester, n/N (%)	Senior-level requester, n/N (%)						
2016	23/1894 (1.2%)	26/658 (4.0%)	49/508 (9.6%)	98/3060 (3.2%)		84.155 (5)		<.001	
2017	22/1811 (1.2%)	37/648 (5.7%)	60/476 (12.6%)	119/2935 (4.1%)		131.622 (5)		<.001	
2018	38/1772 (2.1%)	44/644 (6.8%)	71/467 (15.2%)	153/2883 (5.3%)		191.04 (5)		<.001	
2019	34/1755 (1.9%)	54/497 (10.9%)	75/415 (18.1%)	163/2667 (6.1%)		147.299 (5)		<.001	

## Discussion

### Principal Findings

This study aimed to understand the landscape of EMR data utilization for medical research at XWHCMU between 2016 and 2019. In the past 4 years, the use of EMR data for medical research was quite uncommon at the hospital. Though overall utilization rates increased each year, the overall growth rate is slowing, with a frequency of just 0.1 times per person per year in 2019. More so, key research departments at the hospital are not utilizing EMR data for research purposes, while junior-level staff continue to be limited in their ability to use the system.

According to the results of this study, the proportion of hospital staff using EMR data was less than 6% and the frequency of EMR data utilization did not exceed 10 times per 100 researchers in 1 year. Meanwhile, even the top 10 research departments at Xuanwu Hospital reduced the frequency at which they used EMR data for medical research purposes. Current clinical scientific research data collection still heavily relies on semimanual input. In China, the Hospital Information System has continuously improved, with the EMR system accumulating a large amount of valuable health care data. According to the Annual Report on the Status of Chinese Hospital Informatization (2018-2019), more than one-fourth of tertiary medical institutions have invested in EMR data utilization for research [26]. Since prospective clinical research is more demanding and difficult to perform, retrospective research is an important means of obtaining clinical evidence. EMR data can be not only used as independent data, but also tied to administrative data for retrospective research [13,16,17], saving both time and money for medical institutions wishing to carry out such research studies with limited resources [18,19]. Thus, steps within the hospital should be taken to promote the awareness of this type of available research data, along with the encouragement to carry our medical research using these systems. Further evaluations are needed to gain a better understanding as to why current medical staff may not be accessing such data or why these trends may be declining.

Although the frequency of data usage has increased significantly (the fixed base ratio growth rate was 66.67%), this was not found to be significant, and a bottleneck was noted in 2019. The reasons for this decline in data utilization over the last 3 years were not analyzed, though further follow-up studies to determine the factors influencing the decisions for EMR data utilization would be beneficial. These studies could examine if the external environment has changed, including policies for utilizing EMR data, mechanisms for data sharing, and procedures for requesting and obtaining data.

This study also found that older more senior professionals at Xuanwu Hospital were more likely to use EMR data compared to younger age groups (*P*<.001). Junior-level staff should be the main force for tapping the value of the EMR data, as they need scientific research achievements to be promoted and younger individuals tend to accept new technologies and new methods faster compared to older populations [49]. In large general hospitals in China, all professional and technical staff are required to have independent scientific research capabilities and publications. However, there is a serious contrast between actual need and actual use of EMR data among junior-level staff, as seen in this study. While this study did not evaluate such contrasts, other research has aimed to identify why such barriers to data access may exist, as noted in the Introduction section of this manuscript. The first issue of data access may be inequality, as bureaucracy has been noted as one of main barriers when using EMR data for research [48]. If this is the case at hospitals in Beijing, it is urgent to establish an equal and open EMR data utilization mechanism. Another potential barrier is whether there is a lack of awareness of the research value of EMR data among younger junior-level staff [50]. Lastly, the EMR data utilization skills of junior-level staff may be insufficient [51,52]. If awareness and skills are indeed lacking, it is required to establish systematic training and technical support services for this group [53,54].

### Limitations

As this study was limited to one hospital in Beijing, China, the results cannot represent the general situation of other medical institutions in China. In addition, due to information confidentiality, more personnel-related information could not be obtained and the included indicators may not be comprehensive. For other factors that may affect the utilization of EMR data, further research is needed.

### Conclusions

This is the first quantitative study considering EMR data utilization for medical research in a hospital in Beijing. It offers unique insights into the frequency of EMR data usage for medical research purposes and who is utilizing such data. The value of using EMR data for research purposes remains understudied. The results of this study also suggest that young doctors may be less exposed or have less reason to access such research methods. More research is needed to quantify to what extent EMR data are utilized across all hospitals in Beijing and how these systems can enhance future studies.

## Appendix

Multimedia Appendix 1 Research performance assessment standard of XuanWu hospital.

## Abbreviations

EHR: electronic health record
EMR: electronic medical record
EMRAM: Electronic Medical Record Adoption Model
HIMSS: Healthcare Information and Management Systems Society
XWHCMU: Xuanwu Hospital, Capital Medical University
