# Supplementary material for: Using Electronic Medical Record Data for Research in a Healthcare Information and Management Systems Society (HIMSS) Analytics Electronic Medical Record Adoption Model (EMRAM) Stage 7 Hospital in Beijing: Cross-sectional Study
Source: JMIR Med Inform. 2021 Aug 3;9(8):e24405. doi: 10.2196/24405 (PMC8371484; doi:10.2196/24405)
Supplement: Multimedia Appendix 1 [file medinform_v9i8e24405_app1.doc]

**Evaluation indicators and standards of scientific research work at XWHCMU**

| **First indicator** | **Full score** | **Secondary indicators** | **Full score** | **Level** | Scoring criteria | **Measured method** |
| --- | --- | --- | --- | --- | --- | --- |
| **Approved scientific research projects** | 80 | Scientific research projects approved this year | 40 | National | 10 points/item | Check relevant scientific research project approval documents, contracts and grant vouchers  Verify the research project category （3-1） |
|  |  |  |  | Provincial | 8 points/item |  |
|  |  |  |  | Bureau level | 5 points/item |  |
|  |  |  |  | Cooperative commissioned project | 3 points/item |  |
|  |  |  |  | Hospital -level and others | 1 points/item |  |
|  |  | Approved research project funding this year  (RMB, Ten thousand yuan) | 25 | National | Funding amount/5 |  |
|  |  |  |  | Provincial | Funding amount/5 |  |
|  |  |  |  | Bureau level | Funding amount/5 |  |
|  |  |  |  | Hospital -level and others | Funding amount/5 |  |
|  |  | Application for scientific research projects this year | 5 | National | Number of items/5×2 | Check the registration record |
|  |  |  |  | Provincial | Number of items/5×1 |  |
|  |  |  |  | Bureau level | Number of items/5×0.5 |  |
|  |  | International clinical trial registration this year | 10 | International clinical trial registration | 5 points/item | The department provides the international clinical trial registration number and the scientific research department verifies and records |
| **Published papers/**  **books** | 50 | Number of academic papers published in SCI/EI journals per capita this year | 18 | Number of SCI papers published per capita | Articles per capita×48 | Check relevant publications or copies  Calculation formula: total number of articles / number of employees with intermediate titles and above  Verify criteria for article publication (3-2) |
|  |  | Impact factors of academic papers published in SCI journals this year | 8 | Average IF worth points | Total IF value / number of articles | The IF value is based on the Journal Citation Report published by SCI that year |
|  |  | Number of academic papers published per capita in domestic core journals this year | 8 | Number of core journal papers published per capita | Articles per capita×5 | Check relevant publications or copies  Calculation formula: total number of articles / number of employees with intermediate titles and above  Verify criteria for article publication (3-2) |
|  |  | Published works this year (including monographs, translations, and teaching materials) | 6 | Editor-in-chief: 6 points/item  Deputy Editor: 4 points/item  Participation: 2 points | If more than one person in a department participates in the same work, the person with the highest score is count | Department provides relevant copies  (Cover, chief editor/deputy editor/editor list) |
|  |  | National standards and health industry standards formulation | 10 |  | Calculated by project:  As the leading unit: 8 points/item  As a participating unit: 4 points/item | Note: Industry consensus does not count |
| **Transformation of scientific research results** | 60 | National Science and Technology Achievement Award |  | First prize | 40 points/item | View award certificate  Verify Science and Technology Achievement Award Category (3-3) |
|  |  |  |  | Second prize | 30 points/item |  |
|  |  | Provincial and Ministerial Science and Technology Achievement Award |  | First prize | 20 points/item |  |
|  |  |  |  | Second prize | 12 points/item |  |
|  |  |  |  | Third prize | 6 points/item |  |
|  |  | Other scientific and technological achievements awards |  | First prize | 12 points/item |  |
|  |  |  |  | Second prize | 60 points/item |  |
|  |  |  |  | Third prize | 3 points/item |  |
|  |  | Patent authorization |  | Patent | 6 points/item | Check patent certificate |
|  |  |  |  | Innovative application | 2 points/item |  |
|  |  | **New drug approved** |  |  | 15 points/item | Check new drug certificate |
|  |  | New device approved |  |  | 5 points/item | Check medical device approval documents and registration certificates |
|  |  | Transformation of scientific research results |  |  | 10 points/item | Check the contract for the transformation of scientific achievements |
| **Academic events** | 20 | Academic seminar | 12 | Host an international conference | 5 points/item | International Conference: Verification and filing by the President’s Office  Domestic meeting: verification and filing by the Scientific Research Management Office  Academic conference category (3-4) |
|  |  |  |  | Host a national conference | 3 points/item |  |
|  |  |  |  | Host Beijing conference | 1 points/item |  |
|  |  | Academic exchange | 8 | Send overseas training personnel | 1 point/person-time for 3 months and above | International Conference: Verification and filing by the President’s Office  Domestic meeting: verification and filing by the Scientific Research Management Office  The department provides relevant meeting invitations, submissions or poster acceptance certificates and other materials |
|  |  |  |  |  | 0.5 points/person-time for 1 month or more, less than 3 months |  |
|  |  |  |  | Invite well-known foreign experts to give lectures | 0.5 points/person |  |
|  |  |  |  | Invite well-known domestic experts to give lectures | 0.3 points/person |  |
|  |  |  |  | International/domestic conference speeches | International: 1 point/person  Domestic: 0.5 points/person |  |
|  |  |  |  | International/domestic conference posters and contributions | International: 0.5 point/person  Domestic: 0.3 points/person |  |

**Introduction to Evaluation Index**

**3-1 Research project category**

# 1. National：National Science and Technology Major Project, National Key Research and Development Project, Special Fund for Technology Innovation Guidance, The National Natural Science Foundation of China, Clinical trial project^*^

* Our hospital is the head unit of clinical trials and meets one of the following conditions：

（1）Innovative varieties supported by the National or Beijing Science and Technology Program (Class 1 of Chemical Drugs, Class 1 of Biological Products, Class 1 of Traditional Chinese Medicine, Class 3 of Medical Devices)；

（2）Innovative Varieties for International Multi-center Research.

**2. Provincial：**Various scientific research projects set up by the Ministry of Education, National Health Commission, and State Administration of Traditional Chinese Medicine; Project of Beijing Science and Technology Commission (including the Municipal Science and Technology Research Plan, the Torch Project local level project, the Spark Program local level project, the Capital Clinical Characteristic Application Research Project, and the New Star of Science and Technology Project), Beijing Municipal Natural Science Foundation, and Clinical Trial project**^*^**

* Our hospital is the head unit of clinical trials and meets one of the following conditions：

（1）Class 1 of chemical drugs, Class 1 of biological products, Class 1 of traditional Chinese medicine;

（2）Medical devices approved by *Special Approval Procedures for Innovative Medical Devices* and Emergency Approval Procedures for Medical Devices issued by the State Food and Drug Administration；

（3）Drugs in the "*List of drugs for children with priority review and approval*" and "*List of children's medicines encouraged to be developed and declared*" issued by the State Food and Drug Administration；

（4）Used to treat rare diseases.

**3. Bureau level：**Beijing Health Commission（including the Capital Health Development Scientific Research Project, “215” High-level Health Technology Talent Training Plan); Beijing hospitals authority（including "Set sail" Plan Clinical Technology Innovation Project, "Green Seedling Program"); Beijing Administration of Tradition Chinese Medicine（including Capital health development research projects of traditional Chinese medicine); Beijing Municipal Education Commission（including science and technology development projects); Talent Introduction Project set up by the Beijing Municipal Bureau of Foreign Experts; various scientific research projects set up by Peking University Medical Department, Capital Medical University, and other universities（except the President’s Fund of Capital Medical University); Clinical trials project**^*^**

*****meets one of the following conditions：

（1）The sponsor is the selected enterprise of "leap forward development project of Beijing Biomedical Industry;"

（2）Quality and efficacy consistency evaluation of generic drugs;

（3）Our hospital is the head unit of the clinical trial；

（4）If meeting conditions 1 and 2, Xuanwu Hospital is a participating unit to carry out clinical trials.

**4. Cooperative commissioned project:**

（1）All kinds of scientific and technological development, scientific services, scientific research, technical consultations, and other projects entrusted by enterprises and institutions, social organizations, and other relevant departments, as well as various projects issued by government departments through unconventional reporting channels. For example, research projects entrusted by enterprises, cooperation projects, or scientific service projects for testing and processing.

（2）Clinical trial project（other phase I-IV drug and medical devices clinical trials）.

**5. Hospital -level and others**

(1) Various hospital level projects set up by the hospital, such as hospital level basic clinical projects, hospital level nursing projects, or hospital level management topics

(2) Other projects entrusted by various societies, associations, and foundations, such as the Wu Jieping foundation established by the Chinese Medical Association;

(3) Clinical trial items (in vitro diagnostic reagents).

**3-2 Criteria for Article Publication**

1**.** Articles published in statistical source journals or SCI journals as the first author

2**.** A single SCI impact factor is greater than or equal to 5.0 points, the part exceeding the score shall be converted into one SCI paper for every 2.5 points;

3**.** Papers published in supplements, special issues, electronic network editions will not be recognized;

4**.** Reviews, case reports (referring to reports of 3 cases and below) and translations included in the Department article completion index (but not recognized in the hospital title evaluation);

5**.** The registration of papers shall be subject to the publication and shall be valid only with the volume, issue, page number, and other relevant information;

6**.** Articles published overseas, Hong Kong, and Macao, along with sponsored Chinese publications and traditional Chinese printed publications are not recognized;

7**.** Journals not found on the website of the State Administration of press, publication, radio, film, and television will not be recognized.

**3-3 Science and Technology Achievement Award Category**

**1. National：**National Natural Science Award, National Science and Technology Progress Award, and National Invention Award;

**2. Provincial：**Beijing Science and Technology Progress Award, Chinese Medical Association Science and Technology Progress Award, Ministry of Education Science and Technology Progress Award;

**3. Others：**Huaxia Science and Technology Progress Award

**3-4 Academic conference category**

**1. International conferences：**Two or more participating countries (excluding home country), three or more foreign guests attended;

**2. National conferences：**5 provinces or more, and the conference size is 100 or more;

**3. Beijing conferences：**More than 3 participating units and meeting size of 50 or more people.
